# Development of a TaqMan^®^ Allelic Discrimination qPCR Assay for Rapid Detection of Equine *CXCL16* Allelic Variants Associated With the Establishment of Long-Term Equine Arteritis Virus Carrier State in Stallions

**DOI:** 10.3389/fgene.2022.871875

**Published:** 2022-04-13

**Authors:** Come J. Thieulent, Mariano Carossino, Udeni B. R. Balasuriya, Kathryn Graves, Ernest Bailey, John Eberth, Igor F. Canisso, Frank M. Andrews, Michael L. Keowen, Yun Young Go

**Affiliations:** ^1^ Louisiana Animal Disease Diagnostic Laboratory and Department of Pathobiological Sciences, School of Veterinary Medicine, Louisiana State University, Baton Rouge, LA, United States; ^2^ Maxwell H. Gluck Equine Research Center, University of Kentucky, Lexington, KY, United States; ^3^ Department of Veterinary Clinical Medicine, College of Veterinary Medicine, University of Illinois Urbana-Champaign, Urbana, IL, United States; ^4^ Equine Health Studies Program, Department of Veterinary Clinical Sciences, School of Veterinary Medicine, Louisiana State University, Baton Rouge, LA, United States; ^5^ Department of Infectious Diseases and Public Health, Jockey Club College of Veterinary Medicine, City University of Hong Kong, Kowloon, Hong Kong SAR, China

**Keywords:** equine arteritis virus, EAV, equine viral arteritis, EVA, C-X-C motif chemokine ligand 16, CXCL16, allelic discrimination, genotyping

## Abstract

Equine arteritis virus (EAV) is the causative agent of equine viral arteritis (EVA), a respiratory, systemic, and reproductive disease of equids. Following natural infection, up to 70% of the infected stallions can remain persistently infected over 1 year (long-term persistent infection [LTPI]) and shed EAV in their semen. Thus, the LTP-infected stallions play a pivotal role in maintaining and perpetuating EAV in the equine population. Previous studies identified equine C-X-C motif chemokine ligand 16 (CXCL16) as a critical host cell factor determining LTPI in the stallion’s reproductive tract. Two alleles (*CXCL16* ^*S*^ and *CXCL16* ^*r*^) were identified in the equine population and correlated with the susceptibility or resistance of a CD3^+^ T cell subpopulation in peripheral blood to *in vitro* EAV infection, respectively. Interestingly, *CXCL16* ^*S*^ has been linked to the establishment of LTPI in stallions, and thus, genotyping stallions based on *CXCL16* ^*S/r*^ would allow identification of those at the highest risk of establishing LTPI. Thus, we developed a TaqMan^®^ allelic discrimination qPCR assay for the genotyping of the equine *CXCL16* gene based on the identification of a single nucleotide polymorphism in position 1,073 based on NCBI gene ID: 100061442 (or position 527 based on Ensembl: ENSECAG00000018406.2) located in exon 2. One hundred and sixty horses from four breeds were screened for the CD3^+^ T cell susceptibility phenotype to EAV infection by flow cytometry and subsequently sequenced to determine *CXCL16* allelic composition. Genotyping by Sanger sequencing determined that all horses with the resistant CD3^+^ T cell phenotype were homozygous for *CXCL16* ^*r*^ while horses with the susceptible CD3^+^ T cell phenotype carried at least one *CXCL16* ^*S*^ allele or homozygous for *CXCL16* ^*S*^. In addition, genotypification with the TaqMan^®^ allelic discrimination qPCR assay showed perfect agreement with Sanger sequencing and flow cytometric analysis. In conclusion, the new TaqMan^®^ allelic discrimination genotyping qPCR assay can be used to screen prepubertal colts for the presence of the *CXCL16* genotype. It is highly recommended that colts that carry the susceptible genotype (*CXCL16* ^*S/S*^ or *CXCL16* ^*S/r*^) are vaccinated against EAV after 6 months of age to prevent the establishment of LTPI carriers following possible natural infection with EAV.

## Introduction

Equine arteritis virus (EAV) is an enveloped, positive-sense, single-stranded RNA virus that belongs to the family *Arteriviridae*, subfamily *Equarterivirinae*, and genus *Alphaarterivirus* in the order *Nidovirales* (Cavanagh, 1997; Kuhn et al., 2016; Brinton et al., 2021). EAV is the causative agent of equine viral arteritis (EVA) in horses, a respiratory and reproductive disease with worldwide distribution. It causes significant economic losses to the horse industry due to abortion storms, neonatal mortality, respiratory disease, and the establishment of long-term persistent infection (LTPI) in stallions. EAV infection can be asymptomatic or associated with a wide range of clinical signs, including dependent edema, conjunctivitis, periorbital or supraorbital edema, respiratory distress, urticaria, and leukopenia (Timoney and McCollum, 1993; McCollum et al., 1995; MacLachlan et al., 1996; Balasuriya et al., 1999; Balasuriya et al., 2007; Go et al., 2012b; Vairo et al., 2012; Balasuriya et al., 2013; Balasuriya, 2014; Campos et al., 2014; Balasuriya et al., 2016; Carossino et al., 2017a). Additionally, EAV infection of pregnant mares can result in abortion or birth of congenitally infected foals that develop a rapidly progressive and ultimately fatal bronchointerstitial pneumonia or pneumoenteric syndrome (Vaala et al., 1992; Del Piero, 2000). Transmission of EAV can occur through both the respiratory and the venereal routes. Following initial exposure, EAV can establish a persistent infection in the reproductive tract of stallions, resulting in continuous shedding of infectious virus in semen (Balasuriya et al., 2013; Balasuriya et al., 2016; Carossino et al., 2017a). About 10–70% of naturally infected stallions with EAV can become persistently infected carriers. The duration of EAV carrier state in stallions can be divided into short-term (i.e., a few weeks, or a few months and up to 1 year) or long term (i.e., continues to shed virus for many years or even the remainder of the animal’s lifetime) (Go et al., 2011; Balasuriya et al., 2013; Campos et al., 2014; Balasuriya and Carossino, 2017; Carossino et al., 2017b). The ampullae, an accessory sex gland present in the stallion reproductive tract, has recently been recognized as the primary site of EAV persistence in long-term persistently infected carrier stallions (Carossino et al., 2017a; Carossino et al., 2019). Carrier stallions do not exhibit clinical signs of disease or impairment of fertility, which guarantees the maintenance and perpetuation of EAV in the equine population between breeding seasons.

Previous studies in our group have demonstrated that EAV can infect a distinct subpopulation of peripheral CD3^+^ T lymphocytes from certain horses *in vitro*, while CD3^+^ T lymphocytes from other horses remain resistant to infection. A subsequent genome-wide association study (GWAS) revealed that the ability of EAV to infect CD3^+^ T lymphocytes *in vitro* (susceptibility phenotype) is strongly associated with a haplotype in the region of equine chromosome 11 (ECA11) (Go et al., 2011; Go et al., 2012a). Further genomic and proteomic analysis demonstrated that the equine C-X-C motif chemokine ligand 16 (*CXCL16*) gene, located in this region within ECA11, acts as a cellular receptor for EAV (Sarkar et al., 2016b). More importantly, it has been demonstrated that the ability of EAV to infect CD3^+^ T lymphocytes and establish long-term carrier status in stallions is strongly associated with four single nucleotide polymorphisms (SNPs) in the exon 2 of the *CXCL16* gene (A_1073→_T_1073_; G_1099→_C_1099_; T_1102→_A/G_1102_; G_1108→_A_1108_; based on the annotation for the NCBI equine reference *CXCL16* [gene ID: 100061442] sequence or A_527→_T_527_; G_553→_C_553_; T_556→_A/G_556_; G_562→_A_562_; based on the annotation for the Ensembl equine reference *CXCL16* [ENSECAG00000018406.2] sequence), coding for the susceptibility allele, namely *CXCL16* ^*S*^ (Sarkar et al., 2016a). It has been demonstrated that CXCL16S acts as a cellular receptor for EAV while CXCL16R, coded by the resistance allele *CXCL16* ^*r*^, does not. Concurrently, stallions that are either homozygous or heterozygous for the susceptibility allele (*CXCL16* ^*S/S*^, *CXCL16* ^*S/r*^) can become EAV LTPI carriers, while the stallions that are homozygous for the resistant allele (*CXCL16* ^*r/r*^) do not become carriers or they will be only for a limited period [i.e., short-term carriers or shedders (<1 year)] (Carossino et al., 2017a). The presence of the susceptible genotype (*CXCL16* ^*S*^) differs between breeds, as does EAV seroprevalence (Go et al., 2011; Sarkar et al., 2016a; Socha et al., 2020). A high percentage of Standardbred, Saddlebred, Wielkopolska breed, Polish coldblood, and Silesian breed horses have been shown to carry the susceptible genotype. In contrast, the *CXCL16* ^*r*^ homozygous genotype is most often present in Quarter Horses, Thoroughbreds, Arabian horses, Hucul horses, and Malopolska horses. CXCL16 is, therefore, a critical host factor in the establishment and maintenance of EAV LTPI in the stallion and serves as a “hub” gene likely driving a specific transcriptional network for the establishment of LTPI in the ampullae (Carossino et al., 2019).

Determination of the *CXCL16* genotype in stallions is critical to identify those at greatest risk of becoming LTPI EAV carriers and assist with targeted vaccination practices to prevent the occurrence of the carrier state. In this study, we developed a TaqMan^®^ allelic discrimination qPCR assay for rapid and precise genotyping of the *CXCL16* gene. This allelic discrimination qPCR assay was validated on 160 horses previously sequenced for the *CXCL16* gene, showing perfect agreement with conventional Sanger sequencing and flow cytometric analysis of CD3^+^ T lymphocyte’s phenotype.

## Materials and Methods

### Ethics Approval and Consent to Participate

This study was performed in strict accordance with the recommendations in the Guide for the Care and Use of Laboratory Animals of the National Institutes of Health. The Institutional Animal Care and Use Committee (IACUC) at the University of Kentucky, Lexington, KY and Louisiana State University, Baton Rouge, LA approved protocol numbers 2011-0888 and 18-122, respectively. The explicit owner informed consent for the inclusion of animals in this study was not stated.

### Animals

A total of 160 horses from four different breeds, American Saddlebred (SB), Quarter Horse (QH), Standardbred (STB), and Thoroughbred (TB), were randomly selected for this study (*n* = 40 of each breed) (Sarkar et al., 2016a). These horses were randomly selected from farms in central Kentucky.

### Virus and Antibodies

The virulent Bucyrus strain of EAV (VBS; ATCC, Manassas, VA, Cat# VR-796) was used at a multiplicity of infection (MOI) of two for *in vitro* infection of PBMCs. The equine CD3 specific monoclonal antibody (MAb), UC F6G, kindly provided by Jeff Stott, University of California, Davis, was used as the primary antibody. The R-phycoerythrin (R-PE)-conjugated F (ab')_2_ fragment of goat anti-mouse IgG1 (SouthernBiotech, Birmingham, AL, Cat# 1,072-09, RRID: AB_2794434) was used as the secondary antibody. Alexa Fluor 488-labeled MAb against the non-structural protein 1 (nsp1; MAb 12A4) was used to detect EAV antigen (Wagner et al., 2003; Go et al., 2010).

### Blood Collection, Isolation of PBMCs, and Flow Cytometry

Flow cytometric analysis of EAV infected equine peripheral blood mononuclear cells (PBMCs) was performed as previously described (Go et al., 2010; Go et al., 2011). Briefly, blood was collected from each horse using Vacutainer tubes containing 15% EDTA solution (Kendall Healthcare, Mansfield, MA). Next, PBMCs were isolated from the buffy-coat fraction by centrifugation using Ficoll-Plaque Plus (Amersham Biosciences, Piscataway, NJ) and infected with EAV VBS strain for 36 h. The susceptible or resistant phenotype of each animal was defined by dual-color flow cytometric analysis of *in vitro* EAV-infected CD3^+^ T lymphocytes. Flow cytometry data were collected using a BD FACS Calibur™ flow cytometer with approximately 100,000 events and analyzed with BD CellQuest Pro (RRID: SCR_014489) and FlowJo (RRID: SCR_008520) version 10 software.

### DNA Extraction

Genomic DNA (gDNA) was obtained from 2×10^7^ PBMCs of each horse by using the Gentra Puregene Blood kit (Qiagen, Valencia, CA, Cat #158389), following the manufacturer’s instructions. The gDNA concentration was assessed using a NanoDrop 1000 Spectrophotometer (Thermo Scientific NanoDrop 1000 Spectrophotometer, RRID: SCR_020560) at an absorbance ratio of optical density at 260/280 nm by following the manufacturer’s instructions and stored at −20°C until used.

### Equine *CXCL16* Gene Sequencing

**FIGURE 1:**
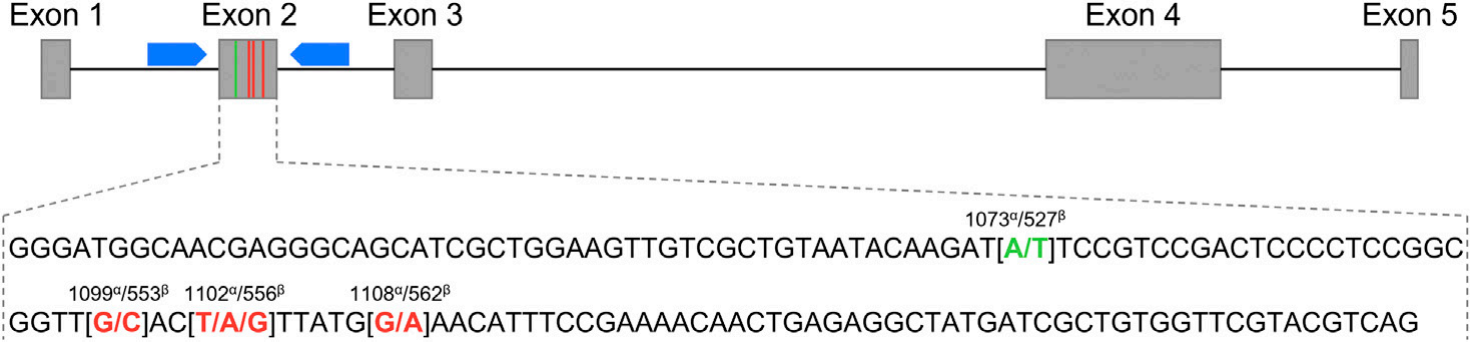
Schematic representation of the *Equus caballus* C-X-C motif chemokine ligand 16 gene (*CXCL16*) organization. The grey boxes represent exons 1 to 5, and the black connecting lines represent introns. The blue pentagons correspond to the primers used for exon 2 sequencing. The exon 2 sequence is depicted below. The SNP at position 1073^α^/527^β^ used for the genotyping qPCR assay is shown in green, and the three other SNPs at positions 1099^α^/553^β^, 1102^α^/556^β^ and 1108^α^/562^β^ are marked in red. ^α^: annotation based on the NCBI *CXCL16* gene ID: 100061442; ^β^: annotation based on Ensembl *CXCL16* gene: ENSECAG00000018406.2.

A polymerase chain reaction (PCR) was performed to amplify a 280 bp sequence encompassing the four SNPs in the *CXCL16* gene as previously described (Sarkar et al., 2016a) (Figure 1). The 18 µl mixture contained 100 ng of gDNA, 0.15 mM of forward primer (5′-CGG​TGG​GTT​GGA​GGC​TAA-3′), 0.15 mM of reverse primer (5′- GAC​CAG​AGA​GGG​TCC​CAG​A-3′) and 10 µl of AmpliTaq Gold™ Fast PCR Master Mix (Applied Biosystems, Waltham, MA, Cat# 4390939). Amplification consisted of the following steps: initial denaturation at 95°C for 10 min and 30 cycles of denaturation at 95°C for 30 s, annealing at 56°C for 30 s and extension at 72°C for 30 s; and a final extension at 72°C for 10 min. Amplicons were then shipped overnight for sequencing to Eurofins Genomics (Louisville, KY, United States). DNA sequences were analyzed using Chromas Lite (Technelysium Pty Ltd., South Brisbane, Australia).

### Equine *CXCL16* Genotyping Assay

A Custom TaqMan^®^ SNP Genotyping Assay (ID: AHX1KFO; Applied Biosystems, Waltham, MA, Cat# 4332077) was designed to amplify a product encompassing the SNP at position 1,073 (A/T) of the equine *CXCL16* gene based on NCBI gene ID: 100061442 (or position 527 based on Ensembl: ENSECAG00000018406.2, respectively). The two fluorogenic MGB probes were designed with VIC and FAM dyes to allow genotyping targeting, respectively, Adenine (*CXCL16* ^*r*^) or Thymine (*CXCL16* ^*S*^) at this position. Real-time PCR was performed on a 25 µl reaction mixture containing 12.5 µl of TaqMan^®^ GTXpress™ Master Mix (Applied Biosystems, Cat# 4401892), 1.25 µl of TaqMan^®^ genotyping assay mix (20×) containing primers and probes, 6.25 µl of DNase-free water, and 5 µl of gDNA diluted at 1 ng/μl in nuclease-free water. Thermal cycling and endpoint analyses were performed on a 7,500 Fast Real-Time PCR System (Life Technologies) using the following conditions: initial DNA polymerase activation step at 95°C for 20 s and 40 cycles of denaturation at 95°C for 3 s and annealing of primers/extension at 60°C for 30 s. The pre-read and post-read were taken at 25°C, according to the TaqMan^®^ GTXpress™ Master Mix protocol.

## Results

### Determination of CD3^+^ T Lymphocyte Phenotypes by Flow Cytometry

**FIGURE 2:**
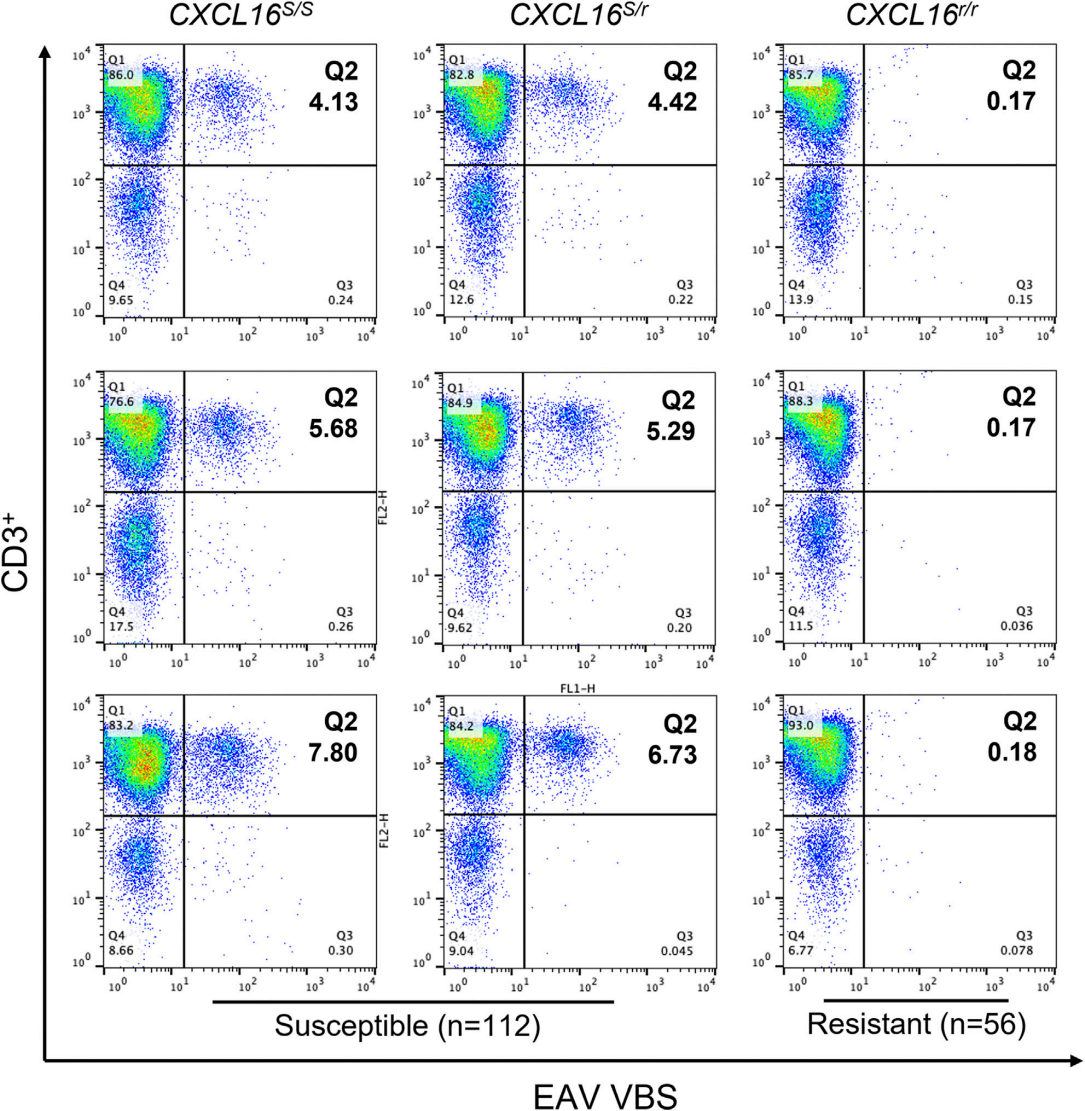
Susceptibility of CD3^+^ T lymphocytes to EAV VBS infection *in vitro*. Equine PBMCs were infected with EAV VBS at an MOI of two and harvested after 36 h in culture. PBMCs were stained with anti-CD3 and anti-nsp1 antibodies, and a dual-color immunofluorescence flow cytometry analysis was performed. Three representative plots corresponding to horses with each of the three *CXCL16* genotypes are shown. Plots were generated using FlowJo version 10 software. The upper right quadrant (Q2) shows the sub-population of CD3^+^ T lymphocytes infected with EAV VBS (CD3^+^ T-cell “susceptible” subpopulation). The numbers in Q2 represent percentages.

Following *in vitro* infection of PBMCs with EAV virulent Bucyrus strain (VBS), a small subpopulation of the CD3^+^ T lymphocytes susceptible to EAV infection (3.9 ± 0.4% to 7.2 ± 0.6%) was identified in 104 horses using flow cytometry (Figure 2; upper right quadrant). Consequently, these horses were classified as having a CD3^+^ T-cell “susceptible” phenotype. In contrast, no CD3^+^ T lymphocytes susceptible to EAV infection were identified in 56 horses, and these were classified as having CD3^+^ T-cell “resistant” phenotype (Table 1).

**TABLE 1:** Determination of *CXCL16* genotypes by DNA sequencing and TaqMan^®^ allelic discrimination qPCR assay and phenotyping *via* flow cytometric analysis of EAV-infected PBMCs (*n* = 160).

CD3^+^ Lymphocytes susceptibility (flow cytometry)	DNA Sequencing			TaqMan^®^ Allelic discrimination qPCR assay			Agreement (%)^a^
	*CXCL16* ^*S/S*^	*CXCL16* ^*S/r*^	*CXCL16* ^*r/r*^	*CXCL16* ^*S/S*^	*CXCL16* ^*S/r*^	*CXCL16* ^*r/r*^	
Susceptible (*n* = 104)	30	74	0	30	74	0	100
Resistant (*n* = 56)	0	0	56	0	0	56	100

a Agreement = (number of correct results for the TaqMan^®^allelic discrimination assay/total of results) × 100.

### *CXCL16* Genotype Determination by Sequencing

A 280 bp sequence encompassing the four SNPs in the exon 2 of the *CXCL16* was obtained by Sanger sequencing (Figure 1) for the 160 horses. Thirty horses were homozygous for the *CXCL16* ^*S*^ allele, 74 were heterozygous (*CXCL16* ^*S/r*^), and 56 horses were homozygous for the *CXCL16* ^*r*^ allele (Table 1). Sequencing data showed that all horses with the susceptible CD3^+^ T lymphocyte phenotype had the *CXCL16* ^*S/S*^ (*n* = 30) or the *CXCL16* ^*S/r*^ (*n* = 74) genotype. In contrast, all the horses with the resistant CD3^+^ T lymphocyte phenotype had the *CXCL16* ^*r/r*^ genotype (*n* = 56).

### Performance of an Allelic Discrimination TaqMan^®^ qPCR Assay for Rapid *CXCL16* Genotyping

**FIGURE 3:**
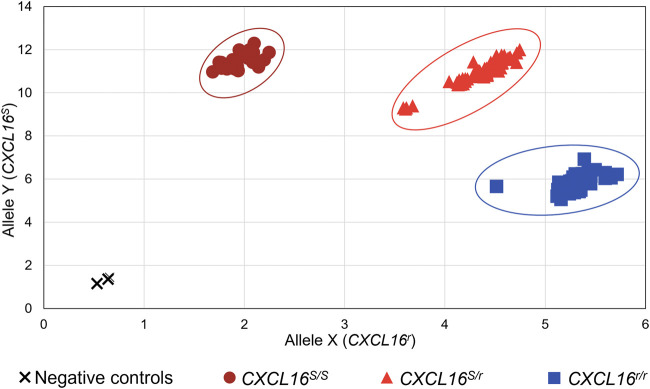
Equine *CXCL16* allelic discrimination assay output and allele determination. The TaqMan^®^ allelic discrimination qPCR assay was used for genotyping *CXCL16* ^*S*^ and *CXCL16* ^*r*^ alleles of 160 horses. The X-axis represents the fluorescence intensity for the *CXCL16* ^*r*^ allele-specific probe labeled with VIC. The Y-axis represents the fluorescence intensity for the *CXCL16* ^*S*^ allele-specific probe labeled with FAM. Dark red circles: homozygous *CXCL16* ^*S/S*^ (*n* = 30); light red triangles: heterozygous *CXCL16* ^*S/r*^ (*n* = 74); blue squares, homozygous *CXCL16* ^*r*/*r*^ (*n* = 56); black cross; negative controls (no DNA). The negatives controls, without gDNA, were located in the bottom left of the graph and were distinct from the positive samples.

The *CXCL16* genotype of the horses was then analyzed using the novel TaqMan^®^ allelic discrimination qPCR assay targeting the A/T SNP at position 1,073 of the equine *CXCL16* gene based on the NCBI gene ID: 100061442 (or position 527 based on Ensembl: ENSECAG00000018406.2, respectively)*,* and results are plotted in Figure 3. Three distinct clusters were graphically obtained, corresponding to horses with the *CXCL16* ^*S/S*^ (*n* = 30), *CXCL16* ^*S/r*^ (*n* = 74), and *CXCL16* ^*r/r*^ (*n* = 56) genotypes. The agreement between the TaqMan^®^ allelic discrimination qPCR assay and Sanger sequencing (gold standard) was 100% (Table 1).

### Distribution of Genotypes by Breed

**FIGURE 4:**
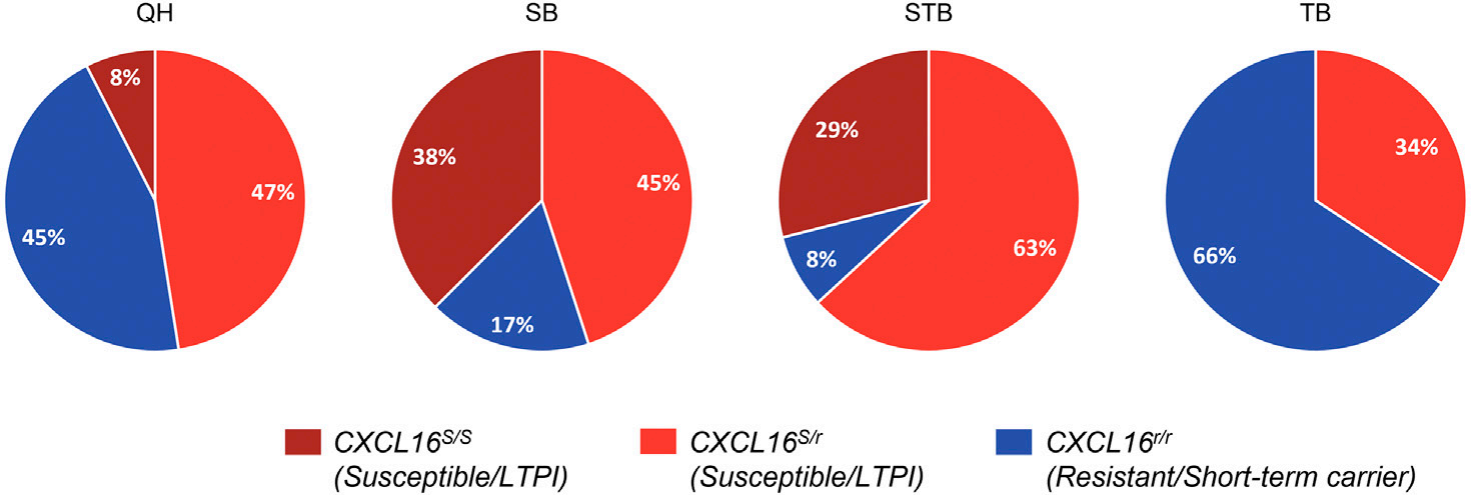
*CXCL16* genotype distribution among diverse horse breeds. Distribution of the three different *CXCL16* genotypes among different horse breeds included in the study (*n* = 40 for each breed). Dark red: *CXCL16* ^*S/S*^; light red: *CXCL16* ^*S*/r^; blue: *CXCL16* ^*r*/*r*^. LTPI: Long-term persistent infection.

Genotype distribution by breed is shown in Figure 4. In concordance with our previous studies, Standardbred (92%) and Saddlebred (83%) horses most frequently carried the *CXCL16* ^*S*^ allele, followed by Quarter Horses (55%) and, lastly, Thoroughbreds, in which the *CXCL16* ^*S*^ allele was detected in approximately one-third (34%) of the population screened.

## Discussion

Single nucleotide polymorphisms (SNPs) are the simplest and most common source of genetic polymorphisms that can cause individual differences in susceptibility to various diseases. Over three million SNPs have been identified in the equine genome (Doan et al., 2012), corresponding to one SNP per 800 bp. Several genomic studies conducted in our laboratory have shown that four SNPs located in the exon 2 of the equine *CXCL16* gene are linked to the establishment of EAV LTPI in the stallion (Sarkar et al., 2016a; Carossino et al., 2017b; Carossino et al., 2019). Indeed, the protein coded by *CXCL16* ^*S*^ was demonstrated to act as a cellular receptor for EAV, while the protein coded by *CXCL16* ^*R*^ does not (Sarkar et al., 2016a; Sarkar et al., 2016b). Therefore, there is a critical need for genotyping the equine CXCL16 gene to rationalize targeted vaccination of stallions with the greatest risk of becoming EAV LTPI carriers and possibly assist in breeding selection. SNP detection using real-time PCR-based assays has been extensively reported (Ehmer et al., 2008; Bowers et al., 2010; Manga and Dvořák, 2010; Kamau et al., 2012; Fedick et al., 2013; Salehi et al., 2015; Heissl et al., 2017). In this study, we have successfully developed a TaqMan^®^ allelic discrimination qPCR assay for genotyping of the equine *CXCL16* gene.

**FIGURE 5:**
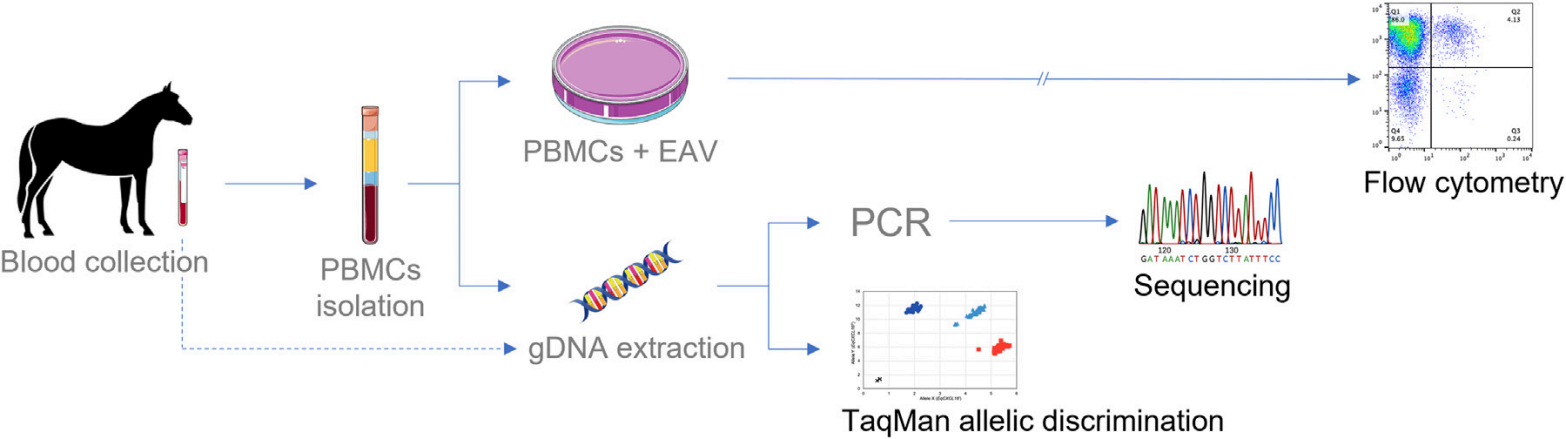
Schematic diagram for testing EAV susceptibility phenotype in CD3^+^ T lymphocytes and genotypification of *CXCL16*. Culturing PBMCs in the presence of EAV followed by flow cytometric analysis is the most extended method for phenotype determination (>36 h). Sanger sequencing of the *CXCL16* gene requires an average of two to three working days for complete analysis. The TaqMan^®^ allelic discrimination qPCR assay is the technique that provides the fastest results, with results in less than 24 h gDNA can be extracted from PBMCs or directly from whole blood.

The four SNPs in exon 2 of *CXCL16* correlate with the presence of a subpopulation of CD3^+^ T lymphocytes in peripheral blood, which is susceptible to EAV infection *in vitro*. Previously, it has been shown that carrier stallions homozygous or heterozygous for the *CXCL16* ^*S*^ allele carry this specific CD3^+^ T lymphocyte subpopulation susceptible to EAV infection *in vitro* (Go et al., 2012a). However, identifying stallions based on this phenotype requires *in vitro* EAV infection of cultured PBMCs followed by dual-color flow cytometric analysis, making this technique time-consuming and technically challenging. In addition, this technique does not differentiate horses that are homozygous from those that are heterozygous for the *CXCL16* ^*S*^ allele (Go et al., 2011). Conventional Sanger sequencing is considered as the gold standard, with high sensitivity for genotypification; however, this methodology is also time-consuming, and occasionally results are affected by inadequate samples, yielding low-quality reads (Sarkar et al., 2016a). In contrast, TaqMan^®^ qPCR assays provide a high-throughput, rapid and straightforward method for genotyping analysis. When comparing the time required to obtain the result, the TaqMan^®^ allelic discrimination qPCR assay is the most effective method, with results obtained in less than a day (Figure 5). Notably, the 100% agreement obtained between Sanger sequencing and TaqMan^®^ assay demonstrates the power and reliability of the latter. This method is a powerful, cost-effective, and rapid screening tool for the genotypification of the *CXCL16* gene. Furthermore, this process is similar to any other real-time PCR assay and can be performed in any molecular laboratory equipped with a real-time thermocycler. In this study, the assay was performed on a 96-well format, but it can easily be converted to a 384-well format for the high-throughput screening of horses.

To conclude, the TaqMan^®^ allelic discrimination qPCR assay described in this study provides a new method for medium to high-throughput genotyping of the equine *CXCL16* alleles with perfect agreement compared to Sanger sequencing allowing accurate identification of stallions at the most significant risk of becoming EAV carriers. Thus, it will assist with targeted vaccination practices with particular emphasis on stallions carrying the *CXCL16* ^*S*^ allele to prevent the occurrence of the carrier state. In addition, this test also opens avenues for selective breeding, which is critical for equine breeding enterprises worldwide and from a disease control perspective.

## Data Availability

The original contributions presented in the study are included in the article/Supplementary Material, further inquiries can be directed to the corresponding author.
